# Molecular adaptations of adipose tissue to 6 weeks of morning fasting vs. daily breakfast consumption in lean and obese adults

**DOI:** 10.1113/JP275113

**Published:** 2017-12-20

**Authors:** Javier T. Gonzalez, Judith D. Richardson, Enhad A. Chowdhury, Francoise Koumanov, Geoffrey D. Holman, Scott Cooper, Dylan Thompson, Kostas Tsintzas, James A. Betts

**Affiliations:** ^1^ Department for Health University of Bath Bath BA2 7AY UK; ^2^ Department of Biology & Biochemistry University of Bath Bath BA2 7AY UK; ^3^ School of Life Sciences Queen's Medical Centre Nottingham NG7 2UH UK

**Keywords:** Nutrition, Adipose tissue, Metabolism

## Abstract

**Key points:**

- In lean individuals, 6 weeks of extended morning fasting increases the expression of genes involved in lipid turnover (*ACADM*) and insulin signalling (*IRS2*) in subcutaneous abdominal adipose tissue.
- In obese individuals, 6 weeks of extended morning fasting increases *IRS2* expression in subcutaneous abdominal adipose tissue.
- The content and activation status of key proteins involved in insulin signalling and glucose transport (GLUT4, Akt1 and Akt2) were unaffected by extended morning fasting. Therefore, any observations of altered adipose tissue insulin sensitivity with extended morning fasting do not necessarily require changes in insulin signalling proximal to Akt.
- Insulin‐stimulated adipose tissue glucose uptake rates are lower in obese *versus* lean individuals, but this difference is abolished when values are normalised to whole‐body fat mass. This suggests a novel hypothesis which proposes that the reduced adipose glucose uptake in obesity is a physiological down‐regulation to prevent excessive *de novo* lipogenesis.

**Abstract:**

This study assessed molecular responses of human subcutaneous abdominal adipose tissue (SCAT) to 6 weeks of morning fasting. Forty‐nine healthy lean (*n* = 29) and obese (*n* = 20) adults provided SCAT biopsies before and after 6 weeks of morning fasting (FAST; 0 kcal until 12.00 h) or daily breakfast consumption (BFAST; ≥700 kcal before 11.00 h). Biopsies were analysed for mRNA levels of selected genes, and GLUT4 and Akt protein content. Basal and insulin‐stimulated Akt activation and tissue glucose uptake rates were also determined. In lean individuals, lipid turnover and insulin signalling genes (*ACADM* and *IRS2*) were up‐regulated with FAST *versus* BFAST (*ACADM*: 1.14 (95% CI: 0.97–1.30) *versus* 0.80 (95% CI: 0.64–0.96), *P = *0.007; *IRS2*: 1.75 (95% CI: 1.33–2.16) *versus* 1.09 (95% CI: 0.67–1.51), *P = *0.03, respectively). In obese individuals, no differential (FAST *versus* BFAST) expression was observed in genes involved in lipid turnover (all *P* > 0.1). GLUT4, Akt protein content and insulin‐stimulated Akt phosphorylation were unaffected by FAST *versus* BFAST in both lean and obese cohorts (all *P* > 0.1). Lower insulin‐stimulated glucose uptake rates in obese *versus* lean individuals were eradicated when normalised to whole‐body fat mass (*P = *0.416). We conclude that morning fasting up‐regulates lipid turnover genes in SCAT of lean individuals. Secondly, altered SCAT insulin sensitivity with morning fasting is unlikely to be explained by signalling proximal to Akt. Finally, lower insulin‐stimulated SCAT glucose uptake rates in obese individuals are proportional to whole‐body fat mass, suggesting a compensatory down‐regulation, presumably to prevent excessive *de novo* lipogenesis in adipose tissue. This trial was registered as ISRCTN31521726.

## Key points

- In lean individuals, 6 weeks of extended morning fasting increases the expression of genes involved in lipid turnover (*ACADM*) and insulin signalling (*IRS2*) in subcutaneous abdominal adipose tissue.
- In obese individuals, 6 weeks of extended morning fasting increases *IRS2* expression in subcutaneous abdominal adipose tissue.
- The content and activation status of key proteins involved in insulin signalling and glucose transport (GLUT4, Akt1 and Akt2) were unaffected by extended morning fasting. Therefore, any observations of altered adipose tissue insulin sensitivity with extended morning fasting do not necessarily require changes in insulin signalling proximal to Akt.
- Insulin‐stimulated adipose tissue glucose uptake rates are lower in obese *versus* lean individuals, but this difference is abolished when values are normalised to whole‐body fat mass. This suggests a novel hypothesis which proposes that the reduced adipose glucose uptake in obesity is a physiological down‐regulation to prevent excessive *de novo* lipogenesis.

## Introduction

Adipose tissue was once considered an inert storage depot, but an understanding of its active roles in metabolic health is gaining momentum (Thompson *et al*. 2012). Adipose tissue is dynamic, buffering daily lipid flux and regulating glucose and lipid metabolism (Garg, 2011; Stanford *et al*. 2015), inflammation, and hormone production (Thompson *et al*. 2012). Importantly, insulin action in adipocytes plays a major role in whole‐body insulin sensitivity (Abel *et al*. 2001) and thus adipose tissue is viewed as a key target for metabolic control.

Subcutaneous adipose tissue is the primary site of storage and release of fatty acids to the systemic circulation (Koutsari & Jensen, 2006). Adipose tissue physiology is highly responsive to acute alterations in energy flux, such as those resulting from fasting, feeding and physical activity (Ruge *et al*. 2009). The acute switch in subcutaneous abdominal adipose tissue (SCAT) metabolism from fasting to feeding results in large changes in glucose and fatty acid flux (Ruge *et al*. 2009). This is partly co‐ordinated by insulin signalling and subsequent translocation of glucose transport proteins and lipoprotein lipase activation (Ruge *et al*. 2009; Chen *et al*. 2017). It is notable that the SCAT response to fasting *versus* feeding is profoundly blunted in obesity. The postprandial increase in glucose and fatty acid uptake by SCAT in obese individuals is less than half that seen in lean individuals (McQuaid *et al*. 2011). This dysregulation of glucose and fatty acid uptake by adipose tissue in obesity is also accompanied by a lower mRNA expression of genes involved in insulin signalling (*IRS2*), glucose transport (*GLUT4*) (Travers *et al*. 2017) and fatty acid metabolism (*ACLS1*, *LPL*, *AGPAT9*, *PLIN*, *HSL*, *PNPL2*, *DGAT1* and *DGAT2*; McQuaid *et al*. 2011; Travers *et al*. 2017), with a concomitant increase in mRNA expression of genes relating to inflammation/cytokine signalling (*IL1RA*, *IL‐18* and *MCP‐1*; Travers *et al*. 2017). While much is known about the distinct metabolic and molecular responses of SCAT to an acute feeding stimulus in both lean and obese individuals, the chronic molecular adaptations of SCAT to modified fasting and feeding patterns have never been assessed in either lean or obese individuals.

The *Bath Breakfast Project* was a randomised controlled trial comparing extended morning fasting (0 kcal until 12.00 h) with daily breakfast consumption (≥700 kcal before 11.00 h) in healthy lean and obese adults (Betts *et al*. 2011). Compared to daily breakfast consumption, extended morning fasting impaired glucose control in SCAT in lean, but not obese, individuals (Betts *et al*. 2014; Chowdhury *et al*. 2016a). Specifically, extended morning fasting in lean individuals increased *in vivo* SCAT interstitial glucose variability in the afternoon and evening (Betts *et al*. 2014) and *ex vivo*, an index of SCAT insulin sensitivity increased from baseline to follow‐up in the breakfast group, but not in the fasting group (Betts *et al*. 2014). Importantly, these findings were not observed in an obese cohort (Chowdhury *et al*. 2016a). Furthermore SCAT from obese individuals displayed quantitatively lower rates of insulin‐stimulated glucose uptake (expressed per milligram lipid) compared to that from lean individuals both at physiological, and supraphysiological insulin concentrations (Chowdhury *et al*. 2016a).

Differences in insulin‐stimulated (physiological and/or maximal) glucose uptake by adipose tissue could be explained by differences in GLUT4 content and/or alterations in insulin signalling and GLUT4 translocation (Tan *et al*. 2015; Travers *et al*. 2015). For example, adipose tissue from individuals who are obese or have type 2 diabetes displays reduced insulin‐stimulated glucose uptake, alongside a 40–85% reduction in GLUT4 protein content (Garvey *et al*. 1991). The lower GLUT4 protein content is thought to be regulated at the mRNA level, as GLUT4 mRNA expression shows a progressive decrease across the range from lean to obese (Travers *et al*. 2015). Akt (PKB) is a key insulin signalling protein that is essential for most metabolic actions of insulin, including phosphorylation of TBC1D4/AS160 leading to GLUT4 translocation (Kane *et al*. 2002; Tan *et al*. 2012). Defects in signalling proximal to Akt have been identified as potential contributors to insulin resistance in various models (Tan *et al*. 2015). We therefore focused on GLUT4 content, Akt signalling and gene expression in SCAT to elucidate the mechanisms that regulate adipose tissue adaptations over 6 weeks of extended morning fasting compared to regular breakfast consumption in lean and obese individuals.

## Methods

### Ethical approval

This study adhered to the standards set by the *Declaration of Helsinki* and the procedures followed were in accordance with the protocol approved by the National Health Service South‐West 3 Research Ethics Committee (10/H0106/13). All participants provided informed written consent prior to participation in the study.

### Experimental design

The *Bath Breakfast Project* is a randomised controlled trial registered with Current Controlled Trials (ISRCTN31521726) and the protocol has been published in full (Betts *et al*. 2011), with trial enrolment, baseline/eligibility testing, allocation and follow‐up all conducted in accordance with CONSORT guidelines (Schulz *et al*. 2010). A CONSORT flow diagram, along with precise details of this protocol and the rationale for our approach/methods have previously been published (Betts *et al*. 2011, 2014). Demographic, anthropometric and physiological characteristics of those who completed the trial are presented in Table 1. This cohort completed intensive laboratory‐based assessments at baseline and we have previously reported resting metabolic rate (via indirect calorimetry from gaseous exchange), anthropometric characteristics (i.e. body mass and DXA‐derived body composition (Discovery W bone densitometer, Hologic Inc., Marlborough, MA, USA)), and some physiological characteristics (specifically, fasting plasma glucose and insulin concentrations; composite Matsuda insulin sensitivity index, adipose tissue glucose uptake rates expressed per milligram lipid) (Betts *et al*. 2014; Chowdhury *et al*. 2015, 2016a, b). We repeat these data here for context in relation to the novel molecular outcomes, and clearly indicate whenever data were previously published.

**Table 1:** Whole‐body characteristics at baseline in lean and obese individuals who donated adipose tissue biopsies^e^

Characteristic		Lean cohort (*n* = 29)	Obese cohort (*n* = 20)	*P*
Age (years)		36 ± 11	43 ± 10	0.033^k^
Female (*n* (%))		21 (64%)	13 (65%)	—
Frequent habitual breakfast consumer^a^ (*n* (%))		26 (79%)	13 (65%)	—
Height (m)		1.71 ± 0.07	1.71 ± 0.10	0.566^l^
Body mass (kg)	PRE	66.2 ± 8.3	98.5 ± 20.1	0.001^k^
Lean mass (DXA^b^)^f^ (kg)	PRE	45.9 ± 8.4	53.5 ± 8.9	0.003^k^
Adipose tissue mass (DXA)^f^ (kg)	PRE	17.3 ± 5.5	37.8 ± 10.0	0.001^k^
Body mass index (kg m^−2^)	PRE	22.6 ± 2.3	33.6 ± 5.0	0.001^k^
Fat mass index^f^ (DXA^c^) (kg m^−2^)	PRE	6.0 ± 2.1	13.3 ± 4.0	0.001^k^
Per cent body fat^f^ (DXA)	PRE	26.4 ± 7.8	40.0 ± 7.3	0.001^l^
Resting metabolic rate^g^ (kcal day^−1^)	PRE	1435 ± 187	1656 ± 296	0.006^l^
Fasting RER	PRE	0.81 ± 0.06	0.83 ± 0.06	0.358^l^
Fasting glucose^f^^,^ h (mmol L^−1^)	PRE	5.35 ± 0.30	5.43 ± 0.38	0.589^l^
Fasting insulin^f^^,^ h (pmol L^−1^)	PRE	22.8 ± 9.6	59.1 ± 27.5	0.001^k^
C‐ISI Matsuda index^d^^,^ i	PRE	9.9 ± 6.7	3.9 ± 1.7	0.001^k^
Fasting NEFA^i^ (mmol L^−1^)	PRE	0.60 ± 0.25	0.53 ± 0.24	0.421^k^

Data are means ± SD unless otherwise stated. *^a^*Defined as the ingestion of ≥50 kcal within 2 h of waking on most days of the week. *^b^*Lean tissue mass excludes bone mineral content. *^c^*DXA‐derived fat mass index obese ranges (Kelly *et al*. 2009) are ≥13 kg m^−2^ (females) and ≥9 kg m^−2^ (males). *^d^*C‐ISI Matsuda index = 1000/√[fasted glucose (mg dL^−1^) × fasted insulin (μIU mL^−1^)] × [mean glucose over 120 min OGTT (mg dL^−1^) × mean insulin over 120 min OGTT (μIU mL^−1^)]. *^e^*Data presented are comparisons of lean and obese participants from Betts *et al*. (2014) and Chowdhury *et al*. (2016a). *^f^n* = 19 for obese. *^g^n* = 28 for lean. *^h^n* = 24 for lean. *^i^n* = 23 for lean and *n* = 16 for obese. *^j^n* = 17 for obese. *^k^*Mann–Whitney test; *^l^*unpaired *t* test. DXA, dual‐energy X‐ray absorptiometry; NEFA, non‐esterified fatty acid.

Participants all met the following inclusion criteria: aged 21–60 years; record of regular menstrual cycle/contraceptive use (if relevant); no anticipated changes in diet and/or physical activity habits during the study period; weight stable (within 2% over past 6 months); non‐shift workers; not pregnant or breastfeeding; and free from any other condition or behaviour deemed either to pose undue personal risk or introduce bias into the experiment.

While all participants in both groups remained in a 10 h overnight fasted state (i.e. 09.00 ± 1 h), a small (∼1 g) subcutaneous adipose tissue biopsy (∼1 g) was sampled from the abdomen, ∼5 cm lateral to the umbilicus, by needle aspiration (14 gauge) after local anaesthesia (4 mL, 1% lidocaine) as previously described (Walhin *et al*. 2013; Travers *et al*. 2015). These were used to provide measures of the post‐absorptive protein content of GLUT4, Akt1 and Akt2, in addition to the mRNA expression of key selected genes involved in adipose tissue metabolism and inflammation. Furthermore, measures of adipose tissue glucose uptake and Akt phosphorylation were performed under basal and insulin‐stimulated conditions.

These measures were followed up 6 weeks later, along with continuous (5 min sampling interval) monitoring of interstitial glucose concentrations via a subcutaneous abdominal catheter (iPro, Medtronic, Watford, United Kingdom) both to document chronic glycaemic responses and to verify compliance, as reported previously (Betts *et al*. 2014; Chowdhury *et al*. 2016a). Eumenorrhoeic women provided baseline samples 2 weeks prior to the start of the 6‐week intervention so that follow‐up samples could be acquired 3–10 days after the onset of menses (i.e. follicular phase). During the 6‐week intervention, participants were randomised (1:1 allocation ratio) into either a group prescribed a self‐selected energy intake of ≥700 kcal before 11.00 h daily, with at least half consumed within 2 h of waking (BFAST group) or a group to extend their overnight fast by abstaining from ingestion of energy‐providing nutrients (i.e. plain water only) until 12.00 h each day (FAST group). The randomisation scheme was generated by the principal investigator (J.A.B.) using a computer‐based random number generator and was stratified according to baseline breakfast habits (block size = 4), with frequent breakfast consumption defined as the ingestion of ≥ 50 kcal within 2 h of waking on most days of the week. Investigators enrolling participants (J.D.R. and E.A.C.) were unaware of these details and independently requested group assignments to prevent deciphering of the allocation sequence. Due to the self‐administered nature of the treatments, it was not possible to blind participants to group allocation. The intervention was applied under free‐living conditions and all other lifestyle choices were allowed to vary naturally. Compliance was confirmed via self‐report and verified via continuous glucose monitoring; data reported herein are therefore only for those individuals for whom baseline and follow‐up measurements are available (i.e. a completers‐only analysis).

### Glucose uptake and protein expression analysis in isolated primary adipocytes

Adipose tissue biopsies were digested with collagenase before determination of d‐[U‐^14^C]glucose uptake as previously described (Foley *et al*. 1983; Kashiwagi *et al*. 1983; Betts *et al*. 2011). The lipid content of adipocytes was determined by extraction and weighing of total lipids of a 50 μL aliquot of 20% adipocytes in suspension. The cell suspension was mixed with 2.7 mL of isopropanol:heptane:H_2_SO_4_ (40:10:1 ratio) followed by the addition of 1.8 mL heptane and 1.0 mL of ddH_2_O before vortexing and centrifugation. A 1.0 mL aliquot of the organic layer was evaporated and the lipid subsequently weighed.

Proteins were separated by SDS‐PAGE and transferred using semidry electrotransfer to a nitrocellulose membrane and were normalised to glyceraldehyde 3‐phosphate dehydrogenase (GAPDH) and per milligram lipid within each sample. Western blotting analysis was performed with the following antibodies: Akt1 (Millipore, Billerica, MA, USA), Akt2 (Millipore), phosphoserine 473 Akt (Cell Signaling Technology, Danvers, MA, USA), GLUT4 (Holman *et al*. 1990), GAPDH (Proteintech, Rosemont, IL, USA). The images were acquired in an EPI Chemi II darkroom (UVP, Upland, CA, USA) and bands quantified using VisionWorks LS analysis software (UVP).

### RNA extraction and quantitative real‐time PCR

Total RNA was extracted by the method of Chomczynski & Sacchi (1987) using TRIzol reagent (Thermo Fisher Scientific, Waltham, MA, USA). Quantification and purity of RNA were assessed using Nanodrop ND‐100 (Thermo Fisher Scientific). Reverse transcription was carried out using 500 ng of total RNA using the SuperScript III cDNA kit (Invitrogen, Paisley, UK). Taqman Low density Custom Array using Micro Fluidic cards (Life Technologies, Thermo Fisher Scientific) was used for the relative quantification of expression of 48 genes as previously described (Tsintzas *et al*. 2013). Each card allowed for eight samples to be run in parallel against 48 Taqman gene expression assay targets that were pre‐loaded into each of the wells on the card. A control sample was loaded in each card to assess reproducibility of data between cards and normalise for variations in the expression of the target genes between cards. Briefly, 50 μL of Taqman Universal PCR master mix (2×) (Life Technologies, Thermo Fisher Scientific) was added to 200 ng RNA equivalent of cDNA into an Eppendorf RNAse‐free tube. RNAse‐free water was added to make the total volume of the reaction mixture up to 100 μL. The reaction mixture was mixed, centrifuged and loaded into one of the fill reservoirs of the Micro Fluidic card. The cards were centrifuged (Multifuge 3 S‐R, Heraeus, Hanau, Germany) and run on a 7900HT Fast Real‐Time PCR System (Life Technologies, Thermo Fisher Scientific). Relative quantification of the genes of interest was performed using the comparative *C*_T_ method. The geometric mean of three housekeeping genes (18S ribosomal RNA (*18S*), *PPIA* and *PGK1*) was used to normalise the data, as previously described (Tsintzas *et al*. 2013, 2017). These genes were selected *a priori*, based on their stability in multiple adipose tissue/adipocyte samples (Neville *et al*. 2011) and this was confirmed in the present study using an algorithm as previously described (Andersen *et al*. 2004). The normalisation to multiple housekeeping genes was used to minimise variations in the expression of individual housekeeping genes (Vandesompele *et al*. 2002). One gene (*UCP1*; uncoupling protein 1) was undetectable and therefore data for 44 genes are presented (Table 2).

**Table 2:** Gene expression assay targets in human subcutaneous abdominal adipose tissue

Gene	Protein/enzyme
*18S rRNA*	18S ribosomal RNA
*ACACA*	Acetyl‐CoA carboxylase alpha
*ACADM*	Medium‐chain acyl‐coenzyme A dehydrogenase
*ADIPOQ*	Adiponectin
*AKT1*	Akt; protein kinase B
*AKT2*	Akt; protein kinase B
*ANGPTL4*	Angiopoietin‐like 4
*CCL2*	Chemokine (C‐C motif) ligand 2
*FABP4*	Fatty acid binding protein 4
*FASN*	Fatty acid synthase
*FOXO1*	Forkhead box protein 01
*G6PD*	Glucose‐6‐phosphate dehydrogenase
*HSD11B1*	11β‐hydroxysteroid dehydrogenase type 1
*IL18*	Interleukin 18
*IL6*	Interleukin 6
*IRS1*	Insulin receptor substrate 1
*IRS2*	Insulin receptor substrate 2
*LEP*	Leptin
*LIPE*	Hormone sensitive lipase
*LPL*	Lipoprotein lipase
*MLXIPL*	Carbohydrate‐response element‐binding protein
*NAMPT*	Nicotinamide phosphoribosyltransferase
*NFKB1*	Nuclear factor NF‐kappa‐B p105 subunit
*NFKB2*	Nuclear factor NF‐kappa‐B p100 subunit
*NR1H2*	Liver X receptor‐beta
*NR1H3*	Liver X receptor‐alpha
*PDK4*	Pyruvate dehydrogenase kinase
*PER2*	PER2
*PGK1*	Phosphoglycerate kinase 1
*PIK3R1*	Phosphatidylinositol 3‐kinase regulatory subunit alpha
*PNPLA2*	Adipose triglyceride lipase/palatin‐like phospholipase domain‐containing protein 2
*PPARA*	Peroxisome proliferator‐activated receptor alpha
*PPARD*	Peroxisome proliferator‐activated receptor delta
*PPARGC1A*	Peroxisome proliferator‐activated receptor gamma coactivator 1‐alpha
*PPARG*	Peroxisome proliferator‐activated receptor gamma
*PPIA*	Peptidylprolyl isomerase A/cyclophilin A/rotamase A
*PRKAA1*	5′‐AMP‐activated protein kinase catalytic subunit alpha‐1
*PRKAA2*	5′‐AMP‐activated protein kinase catalytic subunit alpha‐2
*RETN*	Resistin
*SLC2A4*	GLUT4 (glucose transporter type 4)
*SREBF1*	Sterol regulatory element‐binding transcription factor 1; sterol regulatory element‐binding protein 1
*STAT5A*	Signal transducer and activator of transcription 5A
*STAT5B*	Signal transducer and activator of transcription 5B
*TBC1D4*	AS160 (Akt substrate of 160 kDa); TBC1D4 (TBC1 domain family member 4)
*TNF*	Tumor necrosis factor
*UCP1*	Mitochondrial uncoupling protein 1
*UCP2*	Mitochondrial uncoupling protein 2
*UCP3*	Mitochondrial uncoupling protein 3

### Statistical analyses

**Figure 1:**
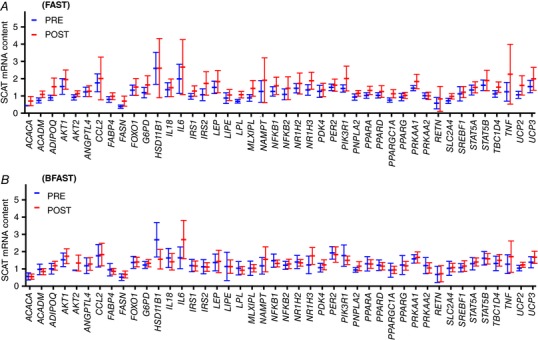
mRNA expression of 44 selected genes in subcutaneous abdominal adipose tissue of lean humans randomised to extended morning fasting (FAST; *n* = 13; *A*) or regular breakfast consumption (BFAST; *n* = 13; *B*) for 6 weeks Values represent the ratios of the mRNA content of target genes to the geometric mean content of housekeeping genes expressed as means ± 95% CI.

**Figure 2:**
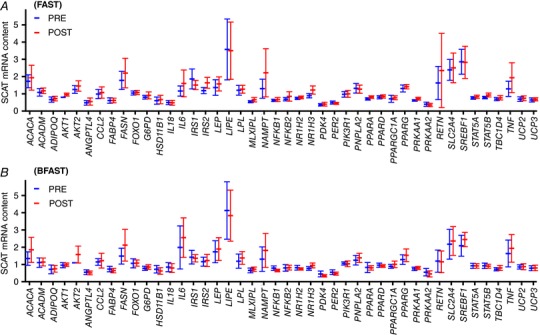
mRNA expression of 44 selected genes in subcutaneous abdominal adipose tissue of obese humans randomised to extended morning fasting (FAST; *n* = 10; *A*) or regular breakfast consumption (BFAST; *n* = 10; *B*) for 6 weeks Values represent the ratios of the mRNA content of target genes to the geometric mean content of housekeeping genes expressed as means ± 95% CI.

**Figure 3:**
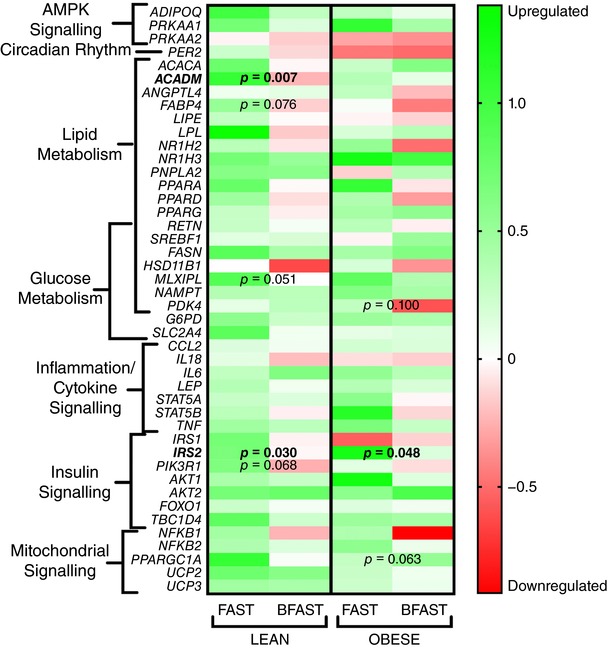
mRNA expression of 44 selected genes in subcutaneous abdominal adipose tissue of lean and obese humans randomised to extended morning fasting (FAST; *n* = 13 lean; *n* = 10 obese) or regular breakfast consumption (BFAST; *n* = 13 lean; *n* = 10 obese) for 6 weeks The intensity of colour represents the effect sizes (Cohen's *d*) of the change from pre‐ to post‐intervention and *P* values represent the FAST *versus* BFAST comparison of mRNA expression levels from ANCOVA with baseline mRNA levels as the covariate.

Statistical analyses were performed using SPSS Statistics v. 24 (IBM Corp., Armonk, NY, USA) and Prism v. 7 (GraphPad Software, San Diego, CA, USA). Values are means with 95% confidence intervals (CI) unless stated otherwise and were checked for normal distribution by the Shapiro–Wilk normality test prior to analysis. mRNA expression is presented as means ± 95% CI in the first instance (Figs 1 and 2), and secondly as effect sizes from baseline to follow‐up in a heat‐map (Fig. 3). This combination was chosen in order to capture both the quantitative mRNA expression and the relative change within the context of variability. The effect size (Cohen's *d*) was calculated as:

d= mean  follow - up -- mean  baseline SD pooled

where:

SD pooled =SD baseline 2+SD follow - up 22

Differences between treatments (breakfast *versus* fasting) were assessed by ANCOVA with baseline scores as the covariate (Bland & Altman, 2015). The phosphorylation status of Akt at basal and physiological insulin concentrations was assessed by ANCOVA with both treatment (breakfast *versus* fasting) and insulin concentration (basal *versus* 50 pm) as fixed factors and baseline scores as the covariate. All *P*‐values have been adjusted for multiple comparisons by Bonferroni correction. Characteristics of lean and obese cohorts at baseline were compared by Student's paired *t* test or Mann–Whitney test as appropriate. Since the *a priori* plan of this study was to assess lean and obese cohorts separately (Betts *et al*. 2011), the responses of lean and obese cohorts to the intervention were not statistically assessed, and therefore inferences of each cohort's responses should be considered separately from one another. Relationships between glucose uptake and GLUT4 protein content were assessed with Pearson's correlation coefficient.

## Results

### Relative post‐absorptive mRNA expression pre‐ and post‐intervention

In lean individuals, the post‐absorptive adipose tissue mRNA expression of genes involved in lipid turnover and the proximal components of the insulin signalling pathway (*ACADM* and *IRS2*) was up‐regulated with extended morning fasting relative to regular breakfast consumption in the lean cohort (*P* = 0.007 and 0.03, respectively; Figs 1 and 3). However, there was no differential (breakfast *versus* fasting) mRNA expression of four key genes further downstream of *PIK3R* in lean individuals (Fig. 3; all *P* > 0.1). The mRNA expression of genes involved in AMPK signalling, inflammation/cytokine signalling, and mitochondrial signalling was not affected by extended morning fasting, compared to regular breakfast consumption (all *P* > 0.1; Fig. 3).

In obese individuals, the post‐absorptive adipose tissue mRNA expression of genes involved in lipid turnover was not affected by extended morning fasting *versus* regular breakfast consumption (all *P* > 0.1; Figs 2 and 3). *IRS2* mRNA expression was increased with extended morning fasting *versus* regular breakfast consumption (*P* = 0.049; Fig. 3); however, genes involved in insulin signalling proximal to *IRS2* displayed no differential regulation by extended morning fasting *versus* regular breakfast consumption in obese individuals (all *P* > 0.1; Fig. 3). The mRNA expression of genes involved in AMPK signalling and inflammation/cytokine signalling was unaffected by extended morning fasting, compared to regular breakfast consumption (all *P* > 0.1; Fig. 3).

### GLUT4, Akt1 and Akt2 protein content

**Figure 4:**
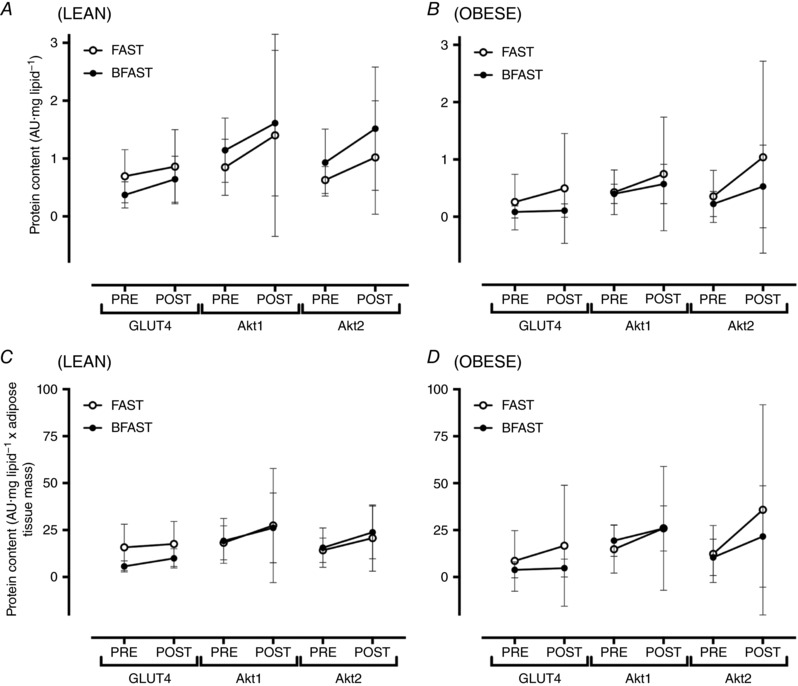
Protein content of glucose transporter 4 (GLUT4), Akt1 and Akt2 in adipose tissue of lean (*A* and *C*) and obese (*B* and *D*) humans randomised to extended morning fasting (FAST; *n* = 5 lean; *n* = 4 obese) or regular breakfast consumption (BFAST; *n* = 4 lean; *n* = 8 obese) for 6 weeks Data are expressed per mg lipid (*A* and *B*) and per mg lipid multiplied by DXA‐derived adipose tissue mass (*C* and *D*) and are presented as means ± 95% CI.

In lean individuals, the protein content of GLUT4, Akt1 and Akt2 was unaffected by extended morning fasting compared to regular breakfast consumption, whether expressed either per milligram lipid, or per milligram lipid multiplied by adipose tissue mass (Fig. 4 *A* and *C*; all *P* > 0.1).

In obese individuals, the protein content of GLUT4, Akt1 and Akt2 was unaffected by extended morning fasting compared to regular breakfast consumption, whether expressed either per milligram lipid or per milligram lipid multiplied by adipose tissue mass (Fig. 4 *B* and *D*; all *P* > 0.1).

### Akt activation

**Figure 5:**
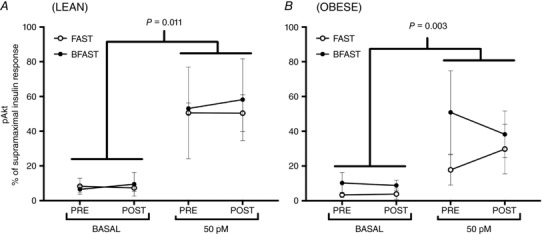
Phosphorylation of Akt expressed as a percentage of maximal phosphorylation (at 20 nm insulin) in isolated adipocytes of lean (*A*) and obese (*B*) humans randomised to extended morning fasting (FAST; *n* = 5 lean; *n* = 4 obese) or regular breakfast consumption (BFAST; *n* = 4 lean; *n* = 8 obese) for 6 weeks Data are presented from measures under basal and physiological (50 pm) insulin concentrations as means ± 95% CI. PRE, pre‐intervention; POST, post‐intervention.

In the lean cohort, Ser 473 phosphorylation of Akt increased up to ∼50% of maximal response with physiological concentrations of insulin (Fig. 5 *A*, *P* = 0.011). But there was no treatment (FAST *versus* BFAST), nor any insulin × treatment interaction effect (*P* = 0.441 and *P* = 0.725, respectively).

In the obese cohort, Ser 473 phosphorylation of Akt increased with physiological concentrations of insulin (Fig. 5 *B*, *P* = 0.003. But there was no treatment (FAST *versus* BFAST), nor any insulin × treatment interaction effect (*P* = 0.627 and *P* = 0.909, respectively).

### Adipose tissue glucose uptake

**Figure 6:**
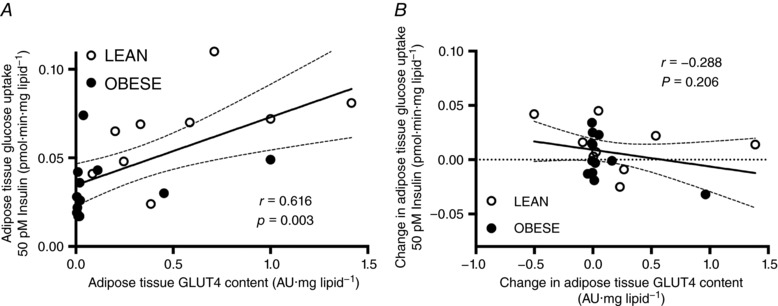
Relationships between protein content of glucose transporter 4 (GLUT4) and glucose uptake in adipose tissue of lean and obese humans randomised to extended morning fasting (FAST; *n* = 5 lean; *n* = 4 obese) or regular breakfast consumption (BFAST; *n* = 4 lean; *n* = 8 obese) for 6 weeks expressed as absolute values at baseline (*A*) or the pre‐ to post‐intervention change (*B*).

At baseline, GLUT4 levels were modestly positively correlated with adipose tissue glucose uptake at physiological insulin concentrations (i.e. 50 pm; Fig. 6 *A*). However, the baseline to follow‐up change in GLUT4 protein content did not correlate with the change in insulin‐stimulated glucose uptake (Fig. 6 *B*).

**Figure 7:**
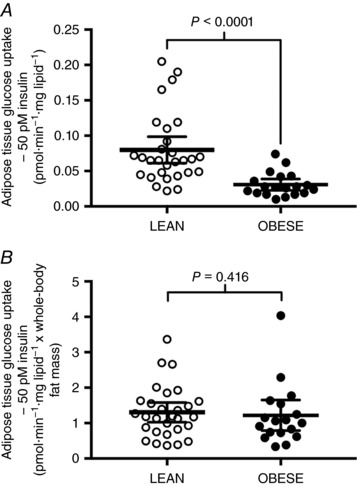
Baseline adipose tissue glucose uptake at physiological concentrations (50 pm) of insulin in lean (*n* = 29) and obese (*n* = 18) humans expressed per mg lipid (*A*), or per mg lipid multiplied by DXA‐derived whole‐body fat mass (*B*) Horizontal lines represent means ± 95% CI. Differences between lean and obese cohorts were compared by Mann–Whitney tests.

At baseline, adipose tissue glucose uptake under physiological concentrations of insulin was ∼2.6‐fold higher in lean compared to obese individuals when expressed per milligram lipid (Fig. 7 *A*; difference between lean and obese: 0.038 pmol min^−1^ (mg lipid)^−1^; *P* < 0.0001), as previously reported without direct comparison between lean and obese cohorts (Chowdhury *et al*. 2016a). Furthermore, adipose tissue glucose uptake expressed per milligram lipid negatively correlated with DXA‐derived whole‐body fat mass (*r* = −0.480, *P* < 0.001). However, once normalised for whole‐body adipose tissue mass, the difference in adipose tissue glucose uptake rates between lean and obese cohorts was abolished (Fig. 7 *B*; difference between lean and obese: 0.148 pmol min^−1^ (mg lipid)^−1^ × adipose tissue mass; *P* = 0.416).

## Discussion

The present work is the first to examine the molecular responses of human adipose tissue to extended morning fasting. Six weeks of extended morning fasting alters the post‐absorptive mRNA expression of a number of metabolic genes in human adipose tissue of lean individuals. In particular, extended morning fasting increases the post‐absorptive expression of genes involved in lipid turnover compared to daily breakfast consumption in lean individuals. Furthermore, important regulators of insulin sensitivity (e.g. *IRS2*) were also up‐regulated by fasting *versus* breakfast consumption in lean individuals. Extended morning fasting did not, however, alter the protein content of GLUT4, Akt1 or Akt2, nor insulin‐induced Akt phosphorylation in either lean or obese individuals. Whilst some previous studies have characterised the response of lean and obese adipose tissue to an acute feeding stimulus (McQuaid *et al*. 2011), the chronic molecular responses of human adipose tissue to different feeding and fasting patterns had never previously been assessed in either lean, nor in obese individuals.

There is strong evidence that the protein content of GLUT4 is an important contributory factor to the reduction in human adipose tissue insulin sensitivity in obesity and type 2 diabetes. GLUT4 content is typically >40% lower in adipose tissue from individuals with obesity and type 2 diabetes, when compared to lean individuals, seemingly regulated at the mRNA level (Garvey *et al*. 1991). In line with this, we observed that the content of GLUT4 positively correlated with adipose tissue glucose uptake at physiological concentrations of insulin, thereby supporting the role of GLUT4 content in adipose tissue insulin sensitivity. These reductions in protein content may therefore contribute to the lower rates of glucose uptake in adipose tissue from obese individuals compared to lean individuals at both physiological and maximal (Chowdhury *et al*. 2016a) insulin‐stimulated conditions.

Whilst GLUT4 content is of importance in explaining the alterations in insulin sensitivity with chronic changes in fat mass, we did not observe changes in protein content of GLUT4, Akt1 or Akt2 with extended morning fasting compared to regular breakfast consumption, at the level of either mRNA or protein. Therefore, changes in the content of these proteins may not be necessary to explain differences in adipose tissue glucose control with extended morning fasting *versus* breakfast consumption in lean individuals, when energy balance is maintained (Betts *et al*. 2014). In support of this, the pre‐ to post‐intervention change in GLUT4 content did not correlate with the change in insulin‐stimulated glucose uptake. The lack of an increase in post‐absorptive GLUT4 and Akt mRNA expression, and in GLUT4, Akt1 and Akt2 protein content, suggests that phosphoprotein signalling leading to increases in GLUT4 translocation and/or activity is likely to be primarily responsible for the alterations in adipose tissue glucose control previously observed with extended morning fasting *versus* breakfast consumption (Betts *et al*. 2014), rather than *de novo* synthesis of proximal signalling proteins or GLUT4 proteins.

In order to assess whether activation of Akt could explain any adaptations in adipose tissue glucose control with altered meal patterns, we assessed the phosphorylation status of Akt in human adipocytes under basal and physiological (50 pm) insulin stimulation *ex vivo*. In neither lean nor obese individuals did extended morning fasting alter insulin‐stimulated Akt phosphorylation compared to regular breakfast consumption. This is consistent with murine data demonstrating that 28 days of high fat diet induced‐insulin resistance in adipose tissue does not coincide with a reduction in Akt phosphorylation or in GLUT4 protein content, but rather with a reduction in AS160 phosphorylation (Tan *et al*. 2015). Therefore, the effects of fasting/feeding patterns on adipose tissue glucose control in lean individuals are likely to be explained by signalling downstream of Akt, but the precise location of this regulation remains to be determined.

The lower insulin‐stimulated adipose tissue glucose uptake per milligram lipid from obese individuals reported previously (Garvey *et al*. 1991; Chowdhury *et al*. 2016a) could represent a physiological down‐regulation of insulin sensitivity. A physiological down‐regulation of adipose tissue non‐esterified fatty acid (NEFA) release in obesity has previously been suggested whereby, with increasing adiposity, the down‐regulation of NEFA release per kilogram of adipose tissue preserves circulating NEFA homeostasis (Karpe *et al*. 2011). Consistent with this, obese participants in the present study did not display elevated circulating plasma NEFA concentrations relative to lean participants, in spite of more than two‐fold greater average fat mass. To explore whether this may also be the case with insulin stimulated glucose uptake, we multiplied the rate of glucose uptake (originally expressed as pmol min^−1^ (mg lipid^−1^)) by DXA‐derived whole‐body adipose tissue mass (kg). Once normalised for whole‐body fat mass, no differences in insulin‐stimulated glucose uptake were detected between lean and obese groups, and the spread of data were remarkably similar between these cohorts. It may therefore be that the reduction in insulin‐stimulated glucose uptake in obese (but otherwise healthy) individuals is a physiological mechanism to limit the absolute rate of glucose uptake by adipose tissue, and thereby constrain the rate of *de novo* lipogenesis. In line with this, others have demonstrated that individuals with obesity have a higher proportion of adipocytes that are refractory (rather than responsive) to insulin‐stimulated glucose uptake *in vitro* (Lizunov *et al*. 2015), and have a lower adipose tissue expression of genes involves in lipogenesis (Roberts *et al*. 2009). However, since our finding was due to exploratory analysis, it should be confirmed in future studies.

In an attempt to explore transcriptional changes that could be chronically altered by changes in fasting and feeding patterns, we also assessed the mRNA expression of selected genes involved in a number of signalling pathways in the post‐absorptive state before and after the intervention. The post‐absorptive expression of a gene involved in lipid turnover (*ACADM*) was up‐regulated with 6 weeks of extended morning fasting relative to daily breakfast consumption, in lean individuals. These responses could be expected since extended morning fasting results in greater rates of whole‐body lipid utilisation (Gonzalez *et al*. 2013) which, both at rest and during low intensity physical activity, is primarily supported by adipose tissue‐derived NEFAs (van Loon *et al*. 2001). Interestingly, these responses were not observed in the obese cohort. Whilst no statistical comparison was made between lean and obese responses to the intervention (due to *a priori* decisions on power), *ACACA* expression tended to respond in the opposite direction (i.e. down‐regulated with extended morning fasting *versus* daily breakfast consumption). It may be that the obese cohort were less metabolically flexible than the lean cohort, and so were mobilising and oxidising lipids to a lesser extent than the lean cohort in the fasted state. Consistent with this, the fasting whole‐body respiratory exchange ratio (RER) of the obese cohort at follow‐up was higher than the lean cohort (lean *versus* obese difference: −0.03, *P* = 0.01). Importantly, this difference in RER is not explained by differences in dietary carbohydrate intake, since daily carbohydrate intake did not substantially differ between lean and obese individuals when expressed in absolute terms (lean: 292 (95% CI: 260–323) g day^−1^; obese: 283 (95% CI: 241–326) g day^−1^, *P* = 0.531), and was therefore higher in lean *versus* obese individuals when expressed relative to body mass (lean: 4.4 (95% CI: 3.9–4.9 g day^−1^ (kg BM)^−1^); obese: 2.9 (95% CI: 2.6–3.3) g day^−1^ (kg BM)^−1^, *P* < 0.001) (Betts *et al*. 2014; Chowdhury *et al*. 2016a).

Lean individuals demonstrated an up‐regulated post‐absorptive mRNA expression of genes involved in the early phases of insulin signalling (e.g. *IRS2*) with extended morning fasting, when compared to daily breakfast consumption. However, such changes were not evident in more distal components of the insulin signalling cascade (e.g. *AKT*, *FOXO1* or *TBC1D4*). Obese individuals also demonstrated an up‐regulation of *IRS2*. These findings are consistent with acute studies in humans, where adipose tissue *IRS2* mRNA expression is up‐regulated with exercise performed in an overnight fasted state, compared to in the fed state (Chen *et al*. 2017) and skeletal muscle *PI3KR1* mRNA expression robustly increases in response to metabolic challenges such as hyperinsulinaemia, hyperglycaemia and exercise (Tsintzas *et al*. 2013, 2017). Since *PI3KR* is known to negatively correlate with proximal insulin signalling under specific metabolic conditions (Barbour *et al*. 2005; Brachmann *et al*. 2005), the trend for an increase in adipose tissue *PI3KR1* expression with extended morning fasting in lean individuals is consistent with reduced adipose tissue glucose control. The finding that *PI3KR1* did not respond to extended morning fasting compared to regular breakfast consumption in obese individuals is likely to be explained by the presence of existing insulin resistance in the adipose tissue of these individuals. This is also consistent with the findings from our previous work demonstrating that extended morning fasting did not alter adipose tissue glucose control in obese individuals (Chowdhury *et al*. 2016a).

It could be considered surprising that mitochondrial genes did not display a differential expression with breakfast consumption *versus* fasting, given that the breakfast group had higher physical activity levels (Betts *et al*. 2014). Physical activity is known to acutely increase mRNA expression of various genes relating to mitochondrial signalling, such as peroxisome proliferator‐activated receptor γ coactivator 1α (PGC1α; *PPARGC1A*) in both skeletal muscle (Stephens *et al*. 2010) and adipose tissue (Chen *et al*. 2017). However, fasting *per se* can increase PGC1α mRNA expression in rodent muscle (de Lange *et al*. 2006) – although data in humans do not fully support this (Tsintzas *et al*. 2006) – and low carbohydrate availability augments the physical activity induced increase in PGC1α mRNA expression in human skeletal muscle (Camera *et al*. 2015). Therefore, it could be that any potential change in PGC1α with our intermittent fasting protocol was offset by a difference in the physical activity patterns between FAST and BFAST groups.

The large (>700 kcal) carbohydrate‐rich breakfast employed in the present study may preclude generalisation to smaller breakfasts differing in composition. However, the rationale for a large carbohydrate‐rich breakfast was to demonstrate proof‐of‐principle with a first study in this area. Furthermore, as an exploratory study of the molecular responses of adipose tissue to regular morning fasting *versus* breakfast consumption, there were insufficient published data to perform a power calculation on the outcome variables described in the present study. Therefore, some outcome variables may be underpowered and future work should aim to confirm and expand upon these findings, especially with breakfasts differing in macronutrient composition.

In conclusion, 6 weeks of extended morning fasting increases the post‐absorptive expression of genes involved in insulin signalling and lipid turnover, compared to daily breakfast consumption in lean individuals. Most of these responses are not observed in obese individuals. However, extended morning fasting did not alter the protein content or insulin‐stimulated phosphorylation of Akt, nor the protein content of GLUT4. Therefore, any potential changes in adipose tissue glucose control with alterations in morning feeding patterns are likely to be due to proteins involved in signalling and GLUT4 translocation downstream of Akt. Finally, adipose tissue from obese individuals displays lower rates of insulin‐stimulated glucose uptake than that from lean individuals, which appears to be proportional to whole‐body fat mass and may represent an adaptive physiological down‐regulation of adipose tissue glucose uptake in obesity, thereby limiting the rate of *de novo* lipid storage.

## Additional information

### Competing interests

None of the authors declare any conflicts of interest in relation to this work.

### Author contributions

This study was conducted at the University of Bath, UK, in collaboration with the University of Nottingham, UK. J.A.B., G.D.H., K.T. and D.T. designed the research; J.A.B., J.D.R., E.A.C. and D.T. conducted the research; G.D.H. and K.T. provided essential reagents and materials and provided access to protein and gene expression assays, respectively; J.T.G., J.A.B., E.A.C., J.D.R. and F.K. analysed data and performed statistical analysis; J.T.G. and J.A.B. wrote the paper and have primary responsibility for final content. All authors read, edited and approved of the final manuscript. All authors also agree to be accountable for all aspects of the work, ensuring that questions related to the accuracy or integrity of any part of the work are appropriately investigated and resolved. Finally, all persons designated as authors qualify for authorship, and all those who qualify for authorship are listed.

### Funding

This project was funded by a grant from the Biotechnology and Biological Sciences Research Council (BB/H008322/1). J.T.G. has also received funding from the European Society for Clinical Nutrition and Metabolism (ESPEN), The Rank Prize Funds, Arla Foods Ingredients and Kenniscentrum, Suiker and Voeding. F.K. is funded by the Medical Research Council (MR/P002927/1).
